# A Curious Case of MRSA Bacteremia and Septic Pulmonary Embolism Secondary to Peripheral Venous Catheter

**DOI:** 10.1155/2021/5544505

**Published:** 2021-04-09

**Authors:** Joshua Twito, Syeda Sahra, Abdullah Jahangir, Neville Mobarakai

**Affiliations:** Staten Island University Hospital, Staten Island, NY 10305, USA

## Abstract

**Background:**

Central venous catheters (CVCs) have been frequently associated with septic thrombophlebitis, bacteremia, and septic emboli. Right-sided infective endocarditis is seen concurrently in patients with septic pulmonary emboli. A case of methicillin-resistant Staphylococcus aureus (MRSA) bacteremia and septic pulmonary emboli secondary to infected peripheral venous catheter (PVC) is reported. Transesophageal echocardiogram (TEE) showed no evidence of infective endocarditis. *Case Presentation*. A 44-year-old female presented to E.R. with left upper extremity pain and swelling at the previously inserted peripheral 18-gauge intravenous catheter site. She also had chest pain, which worsened with inspiration. The patient was found to be in septic shock. Her clinical condition deteriorated acutely. Right upper extremity deep venous thrombosis (DVT) and pulmonary emboli were seen on imaging. Blood cultures grew MRSA. Transthoracic and transesophageal echocardiograms showed no vegetations. The patient responded well to appropriate antibiotics and anticoagulation.

**Conclusion:**

Peripherally inserted catheters are an important portal for pathogen entry and need periodic site assessment and frequent evaluation of their need for insertion. Septic pulmonary emboli can also be seen without any evidence of right-sided infective endocarditis.

## 1. Introduction

Catheter-associated infections are common in hospitalized patients, particularly with central venous catheters in critically ill patients. These infections include but are not limited to soft tissue abscesses, cellulitis, septic phlebitis, pneumonia, and bacteremia. Upper extremity DVTs formed secondary to central venous catheters have been associated with pulmonary emboli [1–4]. Staphylococcus aureus is usually the incriminated organism in catheter-associated infections, and Staphylococcus bacteremia outcomes, if overlooked, can be life-threatening. Right-sided infective endocarditis is a common precedent with septic emboli [5]. The hospital stay and clinical course in these patients are complicated by the need for prolonged antibiotics and anticoagulation [6]. Rare and infrequently, Staphylococcus bacteremia is also seen with peripherally inserted catheters [7]. We present a case of thrombophlebitis from a peripherally inserted catheter leading to Staphylococcus bacteremia and pulmonary embolism without any evidence of infective endocarditis on transthoracic and transesophageal echocardiogram. The patient received appropriate antibiotics and anticoagulation. Physicians should keep the likelihood of staphylococcal bacteremia and showering of septic emboli in mind even with peripherally inserted catheters. Right-sided infective endocarditis is not necessarily always present with septic pulmonary emboli. Observation of hygienic measures while insertion, periodic site assessment, and prompt discontinuation of the catheter when not indicated should be performed to prevent the catheter-associated infections [8].

## 2. Case

A 44-year-old female presented to our emergency department with pain, swelling, and weakness in the left upper extremity. She also complained of pain in her chest, which worsened with inspiration. The patient had noticed having trouble typing on her computer keyboard and an episodic bilateral leg numbness for the past few days. She had a past medical history of recently diagnosed multiple sclerosis, chronic migraines, and seizure disorder (on antiepileptic drug (AED) therapy). The patient was seen two weeks earlier in the Emergency Room with generalized weakness, left arm numbness, and headache. Stroke workup was unremarkable, and the patient was subsequently discharged to follow-up with neurology outpatient.

She denied any other complaints in the time preceding her admission. The patient reported an active lifestyle and denied any tobacco usage, alcohol, or intravenous drug abuse.

On physical examination, the patient displayed a worsening tachycardia, hypotension with a blood pressure of 75/40 mmHg, and a drop in oxygen saturation to 88% at room air. Supplemental oxygen therapy via nasal cannula at a rate of 5 liters per minute improved her oxygen saturation to 99%. A low-dose norepinephrine infusion was started for the hypotension.

The left upper extremity showed tenderness to palpation and swelling where an intravenous catheter was inserted on her last visit to the E.R. two weeks ago. The 18-gauge PVC was inserted by the nursing department in the Emergency Room and was removed 12 hours after the insertion. The patient noticed some redness and tenderness, which had started few days after being discharged and progressively worsened.

The patient received Cefazolin and Vancomycin. Blood work was significant for leukocytosis, mildly elevated blood urea (30 mg/dL), serum glucose of 164 mg/dL, serum lactate 2.5 mg/dL, erythrocyte sedimentation rate (ESR) 49 mm/hr, and a quantitative D-dimer of 3624 ng/mL. Coagulation studies showed activated partial thromboplastin time (aPTT) 25.4 s, and an INR (international normalized ratio) elevated to 1.42 s.

**Figure 1:**
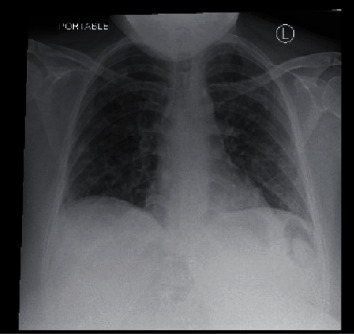
Chest X-ray on arrival showing diffuse bilateral opacities.

**Figure 2:**
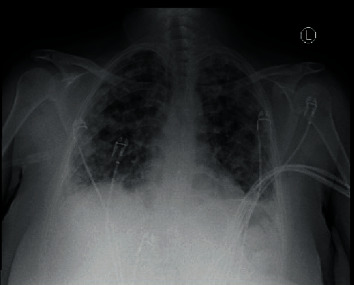
Chest X-ray showing worsening bilateral nodular opacities on hospital day 1.

**Figure 3:**
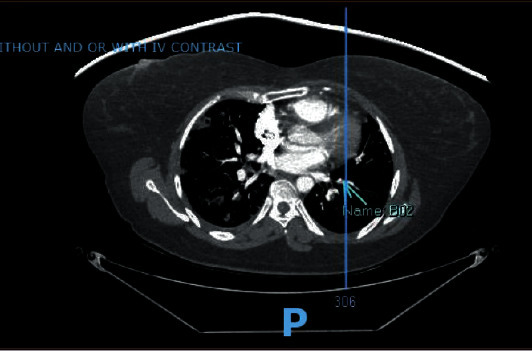
C.T. chest angiogram showing diffuse, multifocal nodular opacities throughout the bilateral lungs, some of which appeared cavitary, with the largest measuring approximately 1.8 cm located in the lower right lobe.

Chest radiograph revealed worsening diffuse nodular opacities bilaterally in the lung fields (Figures 1 and 2). C.T. angiography of the chest with IV contrast revealed changes consistent with septic pulmonary emboli (Figure 3). Left upper extremity duplex ultrasonography revealed acute deep vein thrombosis of the left axillary and brachial veins, thrombosis of the cephalic vein, and a patent basilic vein.

The patient was admitted to the ICU for management of septic shock. Antimicrobial therapy was adjusted to Cefepime and Azithromycin, and therapeutic anticoagulation was started.

On hospital day 2, the left antecubital fossa was erythematous and painful, with purulent fluid expression. Nafcillin and Vancomycin were started based on preliminary cultures growing gram-positive bacteria. Final cultures revealed MRSA bacteremia, and Nafcillin was discontinued. The patient continued to experience chest pain, shortness of breath, and left arm pain with worsening leukocytosis. Transthoracic echocardiogram (TTE) showed no evidence of endocarditis. The patient was found to have septic thrombophlebitis of the brachiocephalic vein on surgical exploration and required excisional debridement and resection of a portion of the brachiocephalic vein. Fungitell, Aspergillus galactomannan assay, and HIV ½ testing were negative.

Vancomycin, Ceftaroline, and Clindamycin were started on the basis of final cultures. Transesophageal echocardiogram (TEE) on hospital day eight did not show any evidence of right-sided endocarditis. Blood cultures were cleared from MRSA on hospital day ten, and we transitioned the anticoagulation to low molecular weight heparin (LMWH). Blood pressure support was weaned off. She got discharged home on intravenous Vancomycin for 6-8 weeks and was reported doing well on follow-up.

## 3. Discussion and Conclusion

Septic pulmonary emboli are characterized by fever, tachycardia, shortness of breath along with nodular opacities, and cavitation on imaging [9]. Septic systemic emboli are seen secondary to left-sided infective endocarditis, and pulmonary emboli are generated secondary to right-sided infective endocarditis [10].

CVC catheter placement is a risk factor for upper extremity DVT [2]. Septic pulmonary embolism follows as its natural consequence. Ultrasonography and color flow doppler are diagnostic modalities for DVTs.

The catheter site serves as a portal for infection. Seeding of community-based and nosocomial pathogens (most commonly Staphylococcus) from the skin has been established to cause bacteremia and right-sided infective endocarditis [5, 11, 12].

There is a substantial emphasis on periodic assessment of centrally placed catheters in critical care units and their prompt removal once they are no longer needed. All emergently placed nonsterile accesses are recommended to be replaced by a sterile catheter as soon as the patient is clinically stable [13]. These ICU protocols have been created and followed after the deadly consequences of catheter-associated infections became well established.

This case is unusual as the patient developed MRSA bacteremia and septic pulmonary emboli from a peripheral venous catheter despite being immunocompetent and having no history of intravenous drug abuse. Another interesting finding was the absence of any right-sided infective endocarditis in this patient confirmed on the transesophageal echocardiogram. Infected PVCs are mostly accompanied by fever, erythema, and tenderness, which resolve with warm compressions and limb elevation. There have been very few bacteremia cases and showering of septic emboli from a peripherally placed venous catheter owing to suppurative thrombophlebitis [14].

A similar case has been reported in the literature recently, but the pathogen was ESBL Klebsiella pneumonia [15]. The authors conclude that peripherally inserted intravenous catheter infections can cause bacteremia and septic emboli. The septic pulmonary emboli are possible without cardiac vegetations as well. Regular assessment of catheter site for erythema and swelling, hygienic measures during insertion, choosing an anatomically favored location for lesser chances of infection (antecubital fossa), and early removal of the catheter can help minimize catheter-associated thrombophlebitis and its complications [8].

## Data Availability

Patient-specific data were obtained from hospital electronic medical records of Northwell Health, and patient identifying information was cropped; individual images are being sent.

## Abbreviations

AEDs: Antiepileptic drugs
CVC: Central venous catheter
DVT: Deep venous thrombosis
ER: Emergency Room
ICU: Intensive care unit
LMWH: Low molecular weight heparin
MSSA: Methicillin-sensitive Staphylococcus aureus
MRSA: Methicillin-resistant Staphylococcus aureus
PVC: Peripheral venous catheter
PTT: Partial thromboplastin time
TTE: Transthoracic echocardiogram
TEE: Transesophageal echocardiogram.
